# Annotations

**Published:** 1896-10-10

**Authors:** 


### Annotations.

### The Hygienic Aspect of Wood Pavement.

The hygienic aspect of wood paving has recently been investigated afresh by MM. Rodet and Nicholas, who have recorded the results of their experiments in the *Lyon Medical.* By dint of the precaution of more thoroughly pulverising the portions of pavement submitted to examination than had been done before, they have been able to demonstrate the presence of a considerably larger number of microbes than had been shown to exist by earlier investigators. In effect they demonstrated that the number of microbic elements in the superficial layers of the wood pavement, after the surface had been washed with water, amounted in one instance to 50,000,000 and in another to 79,360,000 per gramme; while at a depth of five centimetres in the one case they amounted to 51,000 per gramme, and at a depth of six centimetres in the other case to 423,600 per gramme. Only a small proportion, however, of these microbes were liquifying organisms, and in no case did emulsions prepared from them cause any general infection when inoculated into guinea-pigs. The point on which the authors especially insist, and the one which really is of the most importance from a hygienic point of view, is that the microbic infection of wood pavement is not a mere surface affair which can be washed off. The micro-organisms extend into the substance of the wood to a depth of at least a couple of inches in considerable numbers, and exist in enormous multitudes within the superficial layers. From these parts no amount of cleansing can possibly remove them, so that we have in wood paving an uncleanable surface, the wearing away of which must give rise to a form of dust which is very highly charged with microbic life. So far as the investigation goes, however, these microbes, for all their number, do not seem to be of a very injurious nature. What they might be during an epidemic, and how far they might then become associated with more active pathogenic forms, we can only guess by analogy, which in bacteriological matters is not a very safe proceeding; so that, as affairs stand at present, the case against wood pavement as a means of disseminating disease must be held to be "not proven," although open to much suspicion.

### Mr. Hutchinson and Medical Leadership.

Mr. Jonathan Hutchinson, F.R.S., in his address at the opening of the winter session at Owens College, Manchester, the other day, defined for the doctor a position in the modern world which he by no means filled in the ancient or even in the recent centuries. He is to be the "leader," wherever he may happen to be located, in the diffusion of "natural knowledge." Said Mr. Hutchinson: "The medical man, in addition to skill in the application of the existing science of his craft, ought to be ever on the alert for its increase, and ought to be qualified, wherever his lot may fall, to become leader in the extension and diffusion of natural knowledge." Since it has always been one of the lines of the policy of The Hospital to bring about the leadership of the medical profession in the acquisition and diffusion of natural knowledge; and since, in our judgment, the existence and progress of civilisation itself are bound up in the acquisition and diffusion of natural knowledge, we cordially welcome Mr. Hutchinson's very clear and definite utterance on the subject. Let the thoughtful reader consider how the world has progressed since what Bacon called "natural knowledge" began to occupy a chief place in man's activity, and he will immediately admit the absolute imperativeness of future activity in this fruitful field. Let him further ask himself who, in our modern world, except the doctor, is compelled by the very nature of his occupation to be a master in natural knowledge, and he will speedily see that it is the doctor's function alone to occupy the position of leader. The clergyman cannot hold such a position, for "natural knowledge" is in no sense his special study. Still less could the lawyer or the man of business, and for the same reason. The doctor, we again insist, as we have so often insisted before, has now become a chief leader in the advancing army of the world's progress, and from its very commencement The Hospital has recognised this, and has done its utmost to help him to fulfil his comparatively novel, but deeply responsible and ennobling function.

### The Quack Drug Bill.

Ardent total abstainers are fond of telling the public periodically, in the columns of the *Times* and elsewhere, of the enormous amount spent annually by the British public in intoxicating drinks. Mr. Morton Smale, in his opening address at the commencement of the winter session of St. Mary's Medical School the other day, took occasion to give the assembled students a few facts concerning the cost of the quackery which flourishes so extensively in the British Islands. The most striking fact noted by Mr. Smale is the very large cost in money of the quack medicines consumed annually by the British public, amounting as it does to no less a sum than £2,500,000. Of those quack medicines many, as has been analytically demonstrated again and again, have no more actual medicinal potency than plain water. A considerable number, on the other hand, contain ingredients which are deadly poisons, and the indiscriminate use of which, especially among women and children, is decidedly dangerous. Yet, as Mr. Morton Smale pointed out, all these substances are given to the public under the direct "sanction of an enlightened Government," being "protected by Government stamps." This "sanction of the Government," of course, means nothing at all to the educated man or woman. But, unhappily, for every single person who is educated enough to know this, there are at least a hundred who think that a Government stamp must of necessity be a guarantee both of purity and value. And who can blame the average man for supposing that the "sanction of a British Government" ought to be of real tangible worth? As a matter of fact it ought. It is certainly a disgrace to any Government to give its "sanction" to anything of any kind which is not of real merit, and of merit demonstrated to the satisfaction of the Government by persons admittedly competent to make the demonstration. If medicine had but her legitimate influence in the State the whole traffic in patent medicines, as now carried out, would be promptly abolished. We hope the day is not very far distant when the legitimate influence of medicine will be recognised by the State. That day will see the end of a great many brazen and injurious knaveries.

